# O.2.2-9 Initial effects of the “This Girl Can” physical activity and sport mass media campaign in Victoria, Australia

**DOI:** 10.1093/eurpub/ckad133.124

**Published:** 2023-09-11

**Authors:** Adrian Bauman, Nicola McNeil, Matthew Nicholson, Paul O'Halloran, Erica Randle, Emma-Louise Seale, Arthur Stukas

**Affiliations:** Sydney University, Australia; La Trobe University, Australia; La Trobe University, Australia; Monash University, Australia; La Trobe University, Australia; La Trobe University, Australia; La Trobe University, Australia; RMIT, Melbourne, Australia; adrian.bauman@sydney.edu.au; La Trobe University, Australia

## Abstract

**Purpose:**

Mass media campaigns are recommended in the “8 best investments for physical activity”. Despite large investment in the English “This Girl Can” (TGC) campaign since 2015, no independent evaluation data are published. This project reports on the TGC campaign licenced for use in Victoria, Australia from 2018 onwards. This project reports on the impact of the first wave of this media campaign on inactive Australian women’s attitudes and perceptions of physical activity.

**Methods:**

Campaign impact was assessed using serial cohort and independent sample population surveys; two surveys were pre-campaign (Oct-2017 and March-2018), and the post-campaign survey following the first wave of TGC-Victoria mass media (May 2018). Primary analyses used the cohort sample of 818 inactive women followed across all three surveys. We measured campaign awareness and recall, and self-report measures of physical activity behaviour, perceptions of being judged and related psychological measures. Changes in perceptions of being judged and in reported physical activity were assessed in relation to campaign awareness over time.

**Results:**

A third of women recalled the TGC-Victoria campaign. Reported physical activity increased by 0.19days/week following the campaign, especially among women aged over 50years. Feeling judged (3-items, Cronbach alpha=0.91) improved following the campaign (p < 0.01), as did a single-item feeling judged question (p = 0.08), with effect sizes=0.15 and 0.08 respectively. Theory of planned behavior and self-efficacy questions were unchanged but increases noted in self-determination (p < 0.01) and reductions in feeling embarrassed (p < 0.05). No differences were seen in the stage-of-change measure. Campaign awareness was not related to changes in physical activity or feeling judged or embarrassed while being active.

**Conclusions:**

The first year of the TGC-Victoria campaign resulted in good community awareness, and initial decreases in women feeling judged whilst being active, but these did not yet translate into substantial physical activity gains. Further waves of the TGC-V campaign have been conducted to further influence the perception of being judged while being active among inactive Victorian women. This longer term evidence is needed to justify the substantial costs of mass media to promote physical activity.

